# Possible Association of IL-4 VNTR Polymorphism with Susceptibility to Preeclampsia

**DOI:** 10.1155/2014/497031

**Published:** 2014-04-28

**Authors:** Saeedeh Salimi, Milad Mohammadoo-Khorasani, Minoo Yaghmaei, Mojgan Mokhtari, Maryam Moossavi

**Affiliations:** ^1^Cellular and Molecular Research Center, Zahedan University of Medical Sciences, Zahedan 9816743111, Iran; ^2^Department of Clinical Biochemistry, School of Medicine, Zahedan University of Medical Sciences, Zahedan 9816743111, Iran; ^3^Department of Obstetrics and Gynecology, School of Medicine, Zahedan University of Medical Sciences, Zahedan 9816743111, Iran; ^4^Department of Biology, University of Sistan and Baluchestan, Zahedan 9816743111, Iran

## Abstract

Preeclampsia (PE) is a pregnancy-specific disorder that results in maternal mortality and morbidity. Growing evidence indicated that cytokines are involved in the pathogenesis of PE and interleukin-4 VNTR polymorphism could be implicated in altering the PE risk. The aim of this study was to evaluate the possible association between IL-4 VNTR polymorphism and susceptibility to PE in Iranian population for the first time. Genetic polymorphism was evaluated in 192 PE and 186 healthy control women by polymerase chain reaction method. We found that the VNTR polymorphism of IL-4 gene has significantly increased the risk of preeclampsia (RP2/RP1 versus RP1/RP1, OR, 2.8 [95% CI, 1.7 to 8.8]; *P* = 0.0001 and RP2/RP2 versus RP1/RP1; *P* = 0.002). The results showed that carriage of IL-4 VNTR RP2 allele has positive association with preeclampsia susceptibility.

## 1. Introduction 

Preeclampsia (PE) is the most common encountered complication of pregnancy and a public health problem and leads to maternal and neonatal mortality worldwide [1]. This complication is mostly characterized by new onset of hypertension and proteinuria after 20 weeks of gestation. In addition, the PE patients may show signs of renal malfunction, liver disease, and hematological disturbances [2–4]. Despite extensive studies, the basic etiology of PE is still unknown. However, it is confirmed that PE is a multifactorial disorder with a familial predisposition which indicates a genetic contribution [5]. During the pregnancy, there is a shift in immune cells response, T helper 1 (Th1) to T helper 2 (Th2), for favorable implantation, thus inducing maternal tolerance and suppression [6]. In women with PE some of the cytokines released by these cells have been found to be elevated, which could be markers for progression of this syndrome [7]. In this regard, some studies indicated that plasma concentration of proinflammatory cytokines such as TNF-*α*, IL-6, and IL-1*β* which are the main cytokines released by Th1 is higher in patients with PE in comparison to normotensive pregnant women [8–10]. Studies suggested that plasma levels of anti-inflammatory cytokines, including IL-4 as a critical cytokine of Th2, have decreased in PE patients [11, 12]. Interleukin-4 is the main cytokine of T helper 2 lymphocytes, which has a key role in regulation of humoral immune responses [13]. The production of this anti-inflammatory cytokine should increase during pregnancy. Thus, the function of immune system alters during PE. Interleukin-4 (IL-4) gene is mapped within the cytokine gene cluster on chromosome 5q31.1 [14] and there is a 70 bp variable number of tandem repeat (VNTR) polymorphism in its third intron which could change the expression level of IL-4 gene [15]. This VNTR polymorphism contains three alleles: RP1 allele, with three repeats, RP2 allele, with two repeats, and RP3 allele, with four repeats. The frequency of RP1 allele is higher than RP2 alleles. Likewise, RP3 allele is scarce that has been detected in few populations [16].

Although various genetic polymorphisms have been identified as susceptible markers for PE, there is only one published report about the association between IL-4 −590 C > T polymorphism and PE susceptibility. To the best of our knowledge the present study is the first report which aimed to investigate the probable association between IL-4 VNTR polymorphism and the risk of preeclampsia.

## 2. Material and Methods

### 2.1. Population Study

Ethical committee approval was received, and informed consent was obtained from patients and control women before beginning of the study. All individuals were recruited from the Department of Obstetrics and Gynecology of Ali-ebn-Abitaleb educational hospital of Zahedan University of Medical Sciences from 2012 to 2013. This case-control study included 192 women with PE (aged 27.5 ± 7 years) and 186 unrelated healthy controls (aged 26.8 ± 6.4 years).

Preeclampsia was diagnosed according to clinical findings of increased blood pressure (≥140 mmHg systolic or ≥90 mmHg diastolic on 2 or more measurements at least 6 h apart) and proteinuria ≥0.3 g/24 h or ≥+1 on a urine dipstick after 20 weeks of gestation [17]. Exclusion criteria included twin or multiple pregnancies or any evidence of previous medical disease. Women who were affected by systemic, infectious, cardiac, and renal diseases and also systemic lupus erythematosus were excluded. The healthy state of control group was determined by medical history. None of PE patients and healthy controls had any prior history of hypertension. Early-onset PE is usually defined as PE that develops before 34 weeks of gestation, whereas late-onset PE develops at or after 34 weeks of gestation. Severe PE was defined either as severe hypertension (SBP > 160 mmHg or DBP > 110 mmHg) or severe proteinuria (5 g protein in a 24 h urine collection).

### 2.2. Genomic DNA Extraction and Genotyping

The DNA analysis was performed at Cellular and Molecular Research Center (Zahedan, Iran). Blood samples were collected in 2 mL Na-EDTA tubes from patients and healthy controls. Genomic DNA was extracted from peripheral blood leukocytes by salting-out method. The 70 bp VNTR region of IL-4 gene intron 3 was analyzed by polymerase chain reaction (PCR). Two oligonucleotide primers are as follows: forward: 5′AGGCTGAAAGGGGGAAAGC-3′ and reverse: 5′CTGTTCACCTCAACTGCTCC-3′.

PCR reaction was performed in a 25 *μ*L final volume that contained 25 pmol of each primer, 0.1 mmol dNTP (Fermentas, Lithuania), 0.5 *μ*g genomic DNA, 1.5 mM MgCl, 2.5 *μ*L of PCR buffer and 1.5 unit of Taq DNA polymerase (Fermentas, Lithuania) according to the following protocol: initial denaturation at 94°C for 5 min, 30 cycles of denaturation at 94°C for 50 s, annealing at 61°C for 30 s, extension at 72°C for 45 s; and final extension at 72°C for 5 minutes. PCR products were separated by electrophoresis on a 1.5% agarose gel and visualized by ethidium bromide staining.

### 2.3. Statistical Analysis

Data was analyzed using the statistical SPSS v.18 software. The differences between groups were analyzed by independent sample* t*-test, *χ* ^2^ test, or Fisher's exact test, whenever appropriate. Direct gene counting method was used to determine the allele frequency. The genotypes and alleles frequency were compared between PE patients and controls by *χ* ^2^ test and Fisher's exact test. The odds ratio (OR) and 95% confidence intervals (95%CI) were also estimated. Values of *P* < 0.05 were considered statistically significant.

## 3. Results

Demographic data of the PE patients and control group are shown in Table 1. There were no differences in the maternal age and birth weight between two groups. However, PE women had significantly higher systolic and diastolic blood pressures than control group. Moreover, gestational age and primiparity were significantly different between patients and controls (*P* < 0.05). The distribution of three ethnic groups (Balouch, Fars, and Afghan) was significantly different between PE patients and control group (*P* = 0.003), and the risk of PE was 1.7-fold greater in Afghan women in comparison with Balouch and Fars women (OR, 1.7 [95% CI, 1 to 2.8]; *P* = 0.003). There were 44 patients with early-onset PE and 148 patients with late-onset PE. There were also 50 patients with severe PE and 142 patients with mild PE.

The genotype and allele frequencies of IL-4 VNTR polymorphism are shown in Table 2.

The genotypic and allelic frequencies were statistically different between these two groups and the risk of PE was higher in individuals with RP1/RP2 genotype in comparison to those with RP1/RP1 genotype (OR, 2.8 [95% CI, 1.7 to 8.8]; *P* = 0.0001). Furthermore, the frequency of RP2/RP2 genotype in PE patient was more than control group and this difference was significant *P* = 0.002. In addition, the frequency of RP2 allele was significantly higher in PE patients. Therefore, this allele could be a risk factor for PE (OR, 3.1 [95% CI, 1.91 to 4.93]; *P* = 0.0001).

The genotypic and allelic frequencies of IL-4 VNTR polymorphism did not differ between early-onset PE and late-onset PE. In addition, IL-4 VNTR polymorphism was not associated with PE severity. There were not any differences in genotypes and alleles of IL-4 VNTR polymorphism among three ethnic groups (Baluch, Persian, and Afghan).

## 4. Discussion

The immune system has a close relation with pregnancy from implantation to placentation [18] and immunomodulation is prerequisite for successful pregnancy. Hence, the fetus can be protected from maternal immune-cell attacks. Recent studies showed that the altered immune system could lead to PE and several epidemiological findings and animal models support this idea. Immune maladaptation has been reported in PE too [19]. There are multiple factors which develop PE and play key roles in its pathophysiology. Several studies demonstrated that Th1/Th2 balance has changed in patients affected with PE [20–22] and plasma levels of proinflammatory cytokines in PE pregnant women are higher in comparison with normal pregnant women [22–24]. Furthermore, some evidences indicated that serum levels of interlukine-4 in PE patients have decreased. Interleukin-4 belongs to cytokines released by Th2 and acts as an anti-inflammatory cytokine [25, 26].

Recently, the association of several proinflammatory and anti-inflammatory gene polymorphisms such as IL-10 [27], IL-1*β* [28], IL-6 [29], IL-4 [30], IL1-RA [31], and TNF-*α* [32] genes and PE susceptibility has been investigated and in many populations this association was statistically significant.

In the present study we found that RP1/RP2 and RP2/RP2 genotypes and RP2 allele could be risk factors in predisposing to preeclampsia in our population. The RP1/RP2 and RP2/RP2 genotypes were observed more frequently in PE patients than controls (29.1% versus 13.6% and 4.2% versus 0%, resp.). Moreover, the RP2 allele was found more prevalent in preeclampsia patients in comparison with healthy subjects (18.8% versus 6.7%) and may enhance the risk of preeclampsia by 3.1-fold.

IL-4 VNTR polymorphism located in intron 3 of IL-4 gene and could alter messenger ribonucleic acid splicing, which leads to different splice variants [33]. Some studies suggested that RP2/RP2 genotype is associated with low expression of this cytokine [34]. However, another study showed the possible association between RP1 allele and higher expression of the IL-4 [35]. This finding supports this fact that serum level of interlukine-4 in PE patients has decreased.

Up to now, the association of IL-4 VNTR polymorphism with several diseases such as leiomyoma [36], cervical cancer [37], breast cancer [38], end-stage renal disease [39], SLE [40] oral cancer [41], and Kawasaki disease [42] has been investigated. Hsieh et al. indicated lack of association between VNTR polymorphism of IL-4 and leiomyoma disease [36]. However, allelic frequency of RP2 in patients affected with leiomyoma was higher than normal individuals. Shekari et al. showed that genotype frequencies of RP1/RP2 among patients affected with cervical cancer were significantly higher than healthy women [37]. There is only one published report about the association of C > T polymorphism of IL-4 gene with PE which was conducted by Fraser et al. in UK. They found a marked trend for the association between the IL-4 -590 C > T polymorphism and PE [30].

These observations supported the hypothesis that immunological, inflammatory, and anti-inflammatory processes could play key roles in PE development.

Our study suffered from some limitations, for example, low sample size, environmental conditions, and different ethnic groups. For a real understanding of what was said, it is suggested to conduct more extensive studies on larger numbers of people of a community and other ethnic groups from the viewpoint of genetic and environmental factors in order to discuss the role of IL-4VNTR polymorphism in PE pathogenesis more firmly.

In conclusion, we found higher frequency of RP1/RP2 and RP2/RP2 genotypes in comparison to RP1/RP1 genotype in PE patients.

## Figures and Tables

**Table 1:** Demographic characteristics of PE patients and controls.

Variable	Controls	PE patients	P value	OR (95% CI)
	n = 186	*n* = 192		
Maternal age (years)	26.7 ± 6.4	27.5 ± 7	NS	
Gestation age (weeks)	38.5 ± 2.4	37.1 ± 3.5	0.001	
Birth weight	2932 ± 486	2801 ± 23	NS	
SBP	114 ± 9	143.7 ± 22	0.0001	
DBP	71.3 ± 11.5	90.8 ± 13.9	0.0001	
Primiparity *n* (%)	57 (30)	84 (44)	0.003	1.9 (1.2–2.8)
Family history of PE, *n* (%)	61 (33)	77 (40)	NS	
Race, *n* (%)				
Balouch	81 (43.5)	81 (42)		1
Fars	70 (37.6)	52 (27)	0.13	0.7 (0.5–1.1)
Afghan	35 (18.8)	59 (31)	0.003	1.7 (1–2.8)

NS: not significant.

**Table 2:** Genotypes and alleles frequency of the IL-4 VNTR polymorphism in PE patients and controls.

Genotype	Case *N* = 192 (%)	Control *N* = 186 (%)	*P* value	OR (95% CI)
RP1/RP1	128 (66.7)	161 (86.6)	Ref = 1	
RP1/RP2	56 (29.1)	25 (13.6)	0.0001	2.8 (1.7–8.8)
RP2/RP2	8 (4.2)	0 (0)	0.002	—
Allele				
RP1	312 (81.2)	347 (93.3)	Ref = 1	
RP2	72 (18.8)	25 (6.7)	0.0001	3.1 (1.91–4.93)
